# Compression Performance and Failure Analysis of 3D-Printed Carbon Fiber/PLA Composite TPMS Lattice Structures

**DOI:** 10.3390/polym14214595

**Published:** 2022-10-29

**Authors:** Mustafa Saleh, Saqib Anwar, Abdulrahman M. Al-Ahmari, Abdullah Alfaify

**Affiliations:** 1 Industrial Engineering Department, College of Engineering, King Saud University, P.O. Box 800, Riyadh 11421, Saudi Arabia; 2 Raytheon Chair for Systems Engineering (RCSE Chair), King Saud University, P.O. Box 800, Riyadh 11421, Saudi Arabia

**Keywords:** TPMS structures, lattice structures, additive manufacturing, FDM, composite, carbon fiber, PLA, absorption energy

## Abstract

Triply periodic minimum surface (TPMS)-based lattice structures have gained interest for their outstanding capacity to absorb energy, their high load-bearing capacity, and their high surface-to-volume ratio. This study considered three TPMS cell topologies, including Diamond, Gyroid, and Primitive. The FDM process was used to print the lattice structures with two materials: pure polylactic acid (PLA) and carbon fiber-reinforced PLA (PLA + CF). The influence of carbon fiber (CF) incorporation, unit cell type (topologies) and size, and relative density (RD) on mechanical properties and failure patterns were explored comprehensively under uniaxial compression testing. The results demonstrate a change in the compressive modulus (0.09 to 0.47 GPa), compressive strength (2.98 to 13.89 MPa), and specific energy absorption (SEA) (0.14 MJ/m^3^/g to 0.58 MJ/m^3^/g) due to the influence of CF incorporation, cell type and size, and RD. Results indicate that the Diamond structure outperformed both Primitive and Gyroid structures in terms of compressive modulus and strength, and SEA. All the CF-based TPMS structures showed a higher compressive modulus. Compressive strength and energy absorption capacity were both slightly enhanced in most PLA + CF-based Diamond structures. On the contrary, Gyroid and Primitive structures showed better performance for pure PLA-based structures in terms of compression strength and specific absorption energy.

## 1. Introduction

Lattice structures have been intensively studied for their lightweight properties, high energy absorption, and high stiffness-to-weight ratio. Lattice structures are widely used in a variety of applications such as structural components (e.g., in the aerospace [1] and automotive industries [2]), bone-substituting biomaterials or implants [3], impact absorption to protect structures and objects [4], and vibration isolation [5]. This is owing to the fact that lattice structures possess certain combinations of geometrical features, mechanical properties, and physical properties that make them suitable for a wide range of applications (e.g., industrial and medical applications) and allow for the development of structures with improved performance [6,7].

The lattice characteristics are determined by many factors, including the relative density, geometry, constituent material’s properties, and also the manufacturing process, e.g., additive manufacturing (AM) [8,9]. Thus, the functional and mechanical properties of the lattice structures can be tuned to meet the application requirements. As opposed to conventional manufacturing, the advancement of additive manufacturing simplifies the fabrication of lattice structures, allowing for the creation of more designs and controlling relative densities, material compositions, and microstructure. Furthermore, incorporating reinforcement into AM materials certainly uncovers new advanced materials (e.g., composite materials). This allows the manufacturing of composites with enhanced functionality and performance [10], e.g., mechanical [11,12,13,14], biological [3,15,16,17,18,19], electrical [20], thermal [17,20], and chemical performance [18]. A variety of additive manufacturing technologies, including fused deposition modeling (FDM), stereolithography (SLA), digital light processing (DLP), electron beam melting (EBM), and selective laser sintering (SLS), have been used to 3D print lattice structures. Among the AM technologies, FDM has found widespread use in a variety of applications due to its ease of use, low cost, material versatility, and high accuracy [21]. FDM is a prominent AM technology for producing inexpensive custom-made lattice structures.

Various configurations of lattice structures have been designed and fabricated via AM. The most common unit-cell lattice structures are strut-based and triply periodic minimal surfaces (TPMS) [22]. TPMS structures have excellent energy absorption, high load-bearing capacity [23], and a higher surface-to-volume ratio [24] than traditional strut-based lattice structures. The mechanical properties of TPMS lattice structures are unique [25]. Qin et al. [23] demonstrated the potential of 3D-printed TPMS lattice structures with an outstanding energy absorption capability and structural stability.

Despite their capabilities, few studies have investigated the fabrication of TPMS structures, particularly composite materials. Qin et al. [23] studied the impact of material composition on the deformation behavior, compressive modulus, strength, and energy absorption of two lattice structures, cubic and Diamond, FDM 3D-printed from PLA/CaCO_3_ and PLA/TCP composite materials. They stated that adding CaCO_3_ and TCP effectively enhances the compressive modulus and strength of lattice structures. Furthermore, compared to cubic structures, the Diamond structure exhibited improved load-carrying capacity, specific energy absorption, and pore structure stability. Mishra et al. [26] observed a layered pattern of deformation during uniaxial compression testing of an FDM Primitive TPMS lattice made of PLA and ABS materials. The results demonstrate that Primitive structures had a tendency toward catastrophic failure for both materials. Additionally, the influence of different strain rates on the strain energy absorption, modulus of elasticity, and peak strength of both materials was compared. In [27], the mechanical behavior of three TPMS cell topologies, namely, Gyroid, Schwarz Diamond, and Schwarz Primitive, was investigated experimentally and by finite element analysis at different relative densities (10%, 20%, and 30%). The TPMS structures were additively manufactured from PLA material using FDM. They stated that the Diamond structure exhibited the highest mechanical strength, while Primitive structures showed the highest amount of absorbed energy. Abueidda et al. [28] investigated the influence of specimen size and relative density on the stiffness and strength of three polyamide 12 SLS TPMS structures, Primitive, Schoen IWP, and Neovius, when subjected to compression testing. Neovius and IWP showed similar mechanical performance and exhibited more stiffness and strength than Primitive. Shi et al. [29] investigated the compression characteristics, energy absorption capacity, and failure modes of four SLM TPMS lattice structures, namely, Gyroid, Diamond, IW, and Primitive, made of Ti6Al4V, taking relative density into account. Their findings reveal that the cell types and relative densities have an effect on compression performance and energy absorption capacity. In addition, different failure modes were noted for lattice structures. Gyroid, Diamond, and IW structures exhibited a bending–torsional coupling-dominated deformation mode, whereas Primitive structures exhibited a stretch-dominated deformation mode. Spear and Palazotto [30] investigated the influence of different TPMS lattice topology (Diamond, I-WP, and Primitive), cell size, number of cells, and surface thickness on the mechanical properties of SLM lattice structures using INC718 material. The considered factors were found to significantly influence the mechanical characteristics of the lattice structures. The findings indicate the need to consider the design factors and their combination into account when designing a lattice structure, specifically for energy absorption applications.

It is evident from the literature that studies related to FDM TPMS lattice structures are limited. Most of the reported studies focused on singular materials (e.g., PLA [27]), while no reports could be found on exploring the mechanical behavior of composite polymers’ TPMS lattice structures (e.g., PLA + CF). It was also recently highlighted by [23] in 2022 that 3D-printed composite TPMS lattice structures have not been adequately analyzed yet. Due to the fact that material, geometry, and RD determine the lattice structure’s properties, these factors should be comprehensively investigated. Therefore, in this study, an attempt is made to thoroughly study the effect of material composition, geometry, and RD on FDM TPMS lattice structures. In this regard, the TPMS structures were 3D-printed from two materials: pure PLA and carbon fiber-reinforced PLA (PLA + CF). Three TPMS cell topologies, including Primitive, Gyroid, and Diamond, at different cell sizes (6 mm, 8 mm, and 12 mm) and RDs (23% to 44%), were considered. The relative densities of the printed TPMS structures were measured experimentally, and the deviation from the CAD models (designed relative densities) was evaluated using the scanning electron microscope (SEM) images. Uniaxial compression testing was used to characterize and compare the mechanical properties of lattice structures, such as compression curves, compression modulus and strength, and energy absorption capabilities, as well as failure modes.

## 2. Materials and Methods

### 2.1. Materials

In this study, polylactic acid (PLA) polymer with and without carbon fiber materials was used. PLA reinforced with CF (CarbonX™) and pure PLA (ECOMAX^®^) filaments were obtained from 3DXTech, USA. All filaments have a diameter of 1.75 mm and were used as received from 3DXTech. Both filaments are made using Natureworks 4043D PLA biopolymer. The PLA + CF filament incorporates 15% high-modulus carbon fiber in a 4043D PLA matrix. Table 1 shows the chemical composition of PLA and PLA + CF materials. The mechanical properties of the PLA and PLA + CF are presented in Table 2, as received from 3DXTech.

### 2.2. Design and 3D Printing of TPMS Lattices

**Figure 1:**
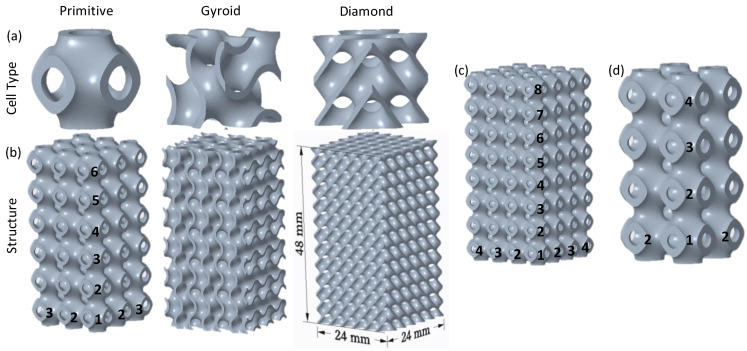
TPMS design: (**a**) unit cell types, (**b**) lattice structures and dimensions, (**c**) Primitive structure with 6 mm cell size (4 × 4 × 8 cells), and (**d**) Primitive structure with 12 mm cell size (2 × 2 × 4 cells). The numbers on the designs represent the number of unit cells that occupied the corresponding design along the x, y, and z directions.

**Figure 2:**
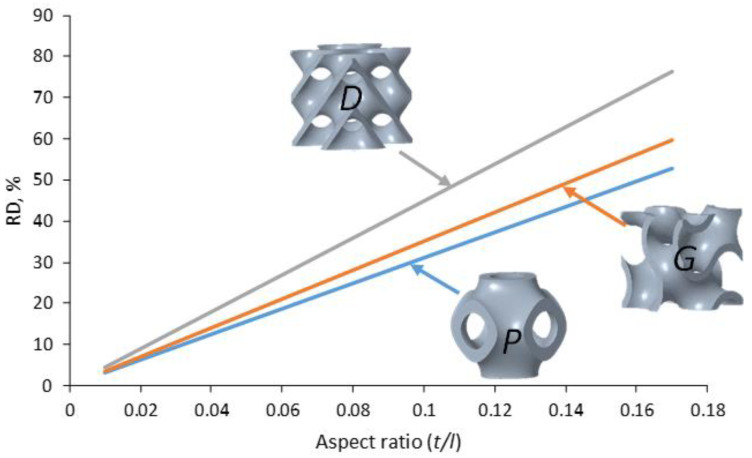
Relative density RD% as a function of *t/l* ratio of Primitive, Gyroid, and Diamond TPMS unit cells.

Three TPMS lattice structures, namely Diamond (D), Gyroid (G), and Primitive (P) were considered. The TPMS unit cells and lattices are shown in Figure 1. TPMS unit cell topology (D, G, and P), relative density (RD) or volume fraction, and unit cell size (*l*) are the parameters that were investigated. The unit cell for all types of TPMS lattice structures was considered cubic, with edge lengths of 6 mm, 8 mm, and 12 mm. These edge lengths (cell sizes) were used to fit the specimen dimensions (24 mm × 24 mm × 48 mm) stated later in this subsection. Relative density is defined as the volume occupied by the lattice structure divided by the overall structure’s volume [33]. In this study, four RDs (23%, 30%, 37%, and 44%) were used, considering the minimum feasible wall thickness that the FDM machine can print. For every unit cell size, the RD or volume fraction was controlled by changing the aspect ratio of the TPMS wall thickness parameter (*t)* to the unit cell size (*t/l)*. The wall thickness parameter *t* controls the TPMS surface thickness [34]. It should be noted that the wall thickness of each unit cell type is varied to meet the required RD, as TPMSs have different surface areas. Figure 2 shows the RD as a function of the aspect ratio of the wall thickness parameter to unit cell size (*t/l)* for the three cell topologies based on the CAD predictions. 

**Figure 3:**
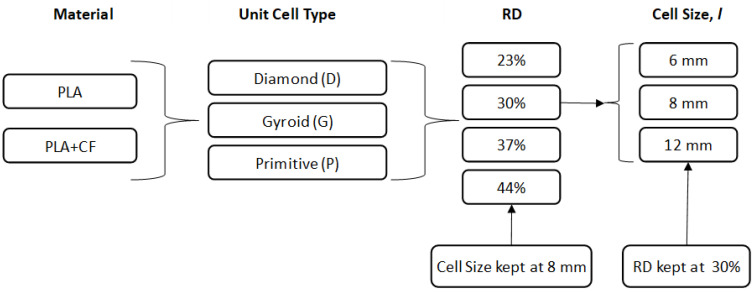
Investigation methodology.

To study the effect of different RDs (23%, 30%, 37%, and 44%) on the compression and energy absorption performance of the TPMS structures, the unit cell size was kept fixed at 8 mm for all cell topologies (P, G, and D), while the wall thickness parameter was changed to achieve the required RD. For example, the wall thickness parameter was set at 0.592 mm, 0.524 mm, and 0.412 mm for Primitive, Gyroid, and Diamond, respectively, to obtain an RD of 23%. Similarly, the RD was kept fixed at 30% to investigate the influence of the three unit cell sizes (6 mm, 8 mm, and 12 mm). This is illustrated in Figure 3.

The lattice samples were designed with dimensions of 24 mm × 24 mm × 48 mm to meet the aspect ratio of the ASTM standard test method for compressive properties of rigid polymer materials (ASTM D695-15) [23,26]. This resulted in a rectangular specimen with 4 × 4 × 8 unit cells (i.e., with four cells along each side of the base and eight cells along the height) in the case of the 6 mm unit cell size (see Figure 1c). Likewise, 3 × 3 × 6 cells (Figure 1b) and 2 × 2 × 4 unit cells (Figure 1d) were filled in the case of the 8 mm and 12 mm unit cell sizes, respectively. A summary of the TPMS lattice design characteristics is presented in Table 3.

The TPMS lattice structures were modeled using CREO Parametric 8.0 software in the STL format. The STL files of the designed lattices were exported from the Creo 8.0 software and imported into PrusaSlicer 2.4.2 for slicing and generating “G-code” files. An original Prusa i3 MK3S+ printer was used to manufacture the TPMS lattices. The printing parameters used are listed in Table 4.

The 3D-printed samples’ dimensions (width, thickness, and length) were measured using a profile projector (Multi-Lens Vertical Lab Profile Projector, VOM-2515). For each dimension, three measurements were taken, and the results were averaged.

### 2.3. Density Measurement

The density of the TPMS structures was measured using Archimedes’ method. The Archimedes method is commonly used to measure the density of solid and porous samples [35]. Due to the formation of air pockets/bubbles during density measurements, which can reduce accuracy, only small cubic samples of 2 × 2 × 2 cells were printed for all designed parameters listed in Table 3 (three samples for each design). Density measurements were conducted on a Shimadzu Analytical Balance (AUW220D) and a universal specific gravity kit (SGK-C, Mineralab).

### 2.4. Morphology Characteristics

The morphology of the printed lattices was evaluated using a tabletop scanning electron microscope (SEM) from JEOL (JCM 6000 plus, Tokyo, Japan). Before imaging, the samples were first platinum-coated using an Auto Fine Coater (JFC-1600, Tokyo, Japan). SEM images were then used to characterize the structural features of the TPMSs and the printing defects that resulted in the actual RDs deviating from the designed RDs.

### 2.5. Mechanical Properties

**Figure 4:**
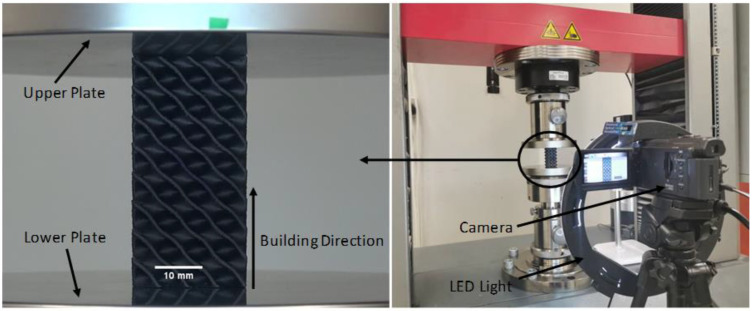
Compression Testing Setup.

The mechanical behaviors of the 3D-printed TPMS lattice structures were characterized by conducting uniaxial compression tests. Compression testing was performed in accordance with the ASTM D695-15 standard. The compression tests were conducted on a Zwick Z100 testing machine with a 100 KN load cell at a constant rate of 1.6 mm/min until 60% strain. Before testing, a preload of 20 N was applied [36]. For each set of parameters in Table 3, three samples were tested, and the results were averaged. Compression testing was conducted along the build direction. Forces and displacements were recorded during the compression testing using the testXpert II software. A digital camera (Sony, HDR-PJ820) was used to record the deformation during the compression testing to analyze the failure mechanisms. The image was recorded at 25 frames per second. The video analysis determined failure mechanisms at a certain strain based on the machine displacement time. The compression testing setup is shown in Figure 4.

The compression modulus (the modulus of elasticity E) was determined by the slope of the tangent line at the initial linear portion of the stress–strain curve by using Zwick’s testXpert II. Peak strength, i.e., the maximum value of the first peak in the stress–strain curve, was used to determine the compressive strength [23,26]. Engineering compressive stress (σ=F/A) was calculated by dividing the force (*F*) by the original cross-sectional area (*A*), whereas the engineering strain $\left(\varepsilon =Disp/L\right)$ was computed by dividing the displacement (*Disp*) by the original length of the sample. The as-built (actual) area and length from the profile projector were used for stress and strain calculations. 

Energy absorption (EA) is defined as the required energy to deform a structure to a strain ε. The energy absorbed per unit volume (MJ/m^3^) by a lattice structure during the compression loading is represented by the area under the stress–strain curve and calculated in Equation (1). The specific energy absorption (SEA) $\left(\mathrm{MJ}/{g\;m}^{3}\right)$ of a structure is one of the most helpful measures for determining a structure’s capability to absorb energy per unit mass. The SEA was calculated by dividing the EA by the mass of the lattice structure (m), as in Equation (2) [37,38,39]. The area under the stress–strain curve of each sample was measured up to 60% ε and was calculated using MATLAB R2022a.

(1) $$\mathrm{EA}=\int_{0}^{\varepsilon }\sigma \left(\varepsilon \right)d\varepsilon$$

(2) SEA=EAm

## 3. Results

### 3.1. Relative Density Characteristics

The density of the TPMS structures was measured using Archimedes’ method. Table 5 shows the relative densities of the TPMS lattice structures under the considered conditions. The variation in the three replicated experimental results is represented by the standard deviation. 

**Figure 5:**
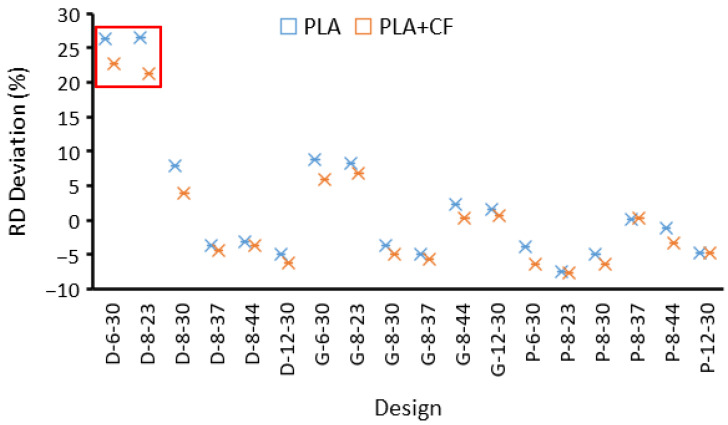
Deviation of the actual RD from the corresponding RD design of the designed lattice structures listed in Table 3. The red box highlights the extreme deviations of the actual RDs from the corresponding CAD designs.

**Figure 6:**
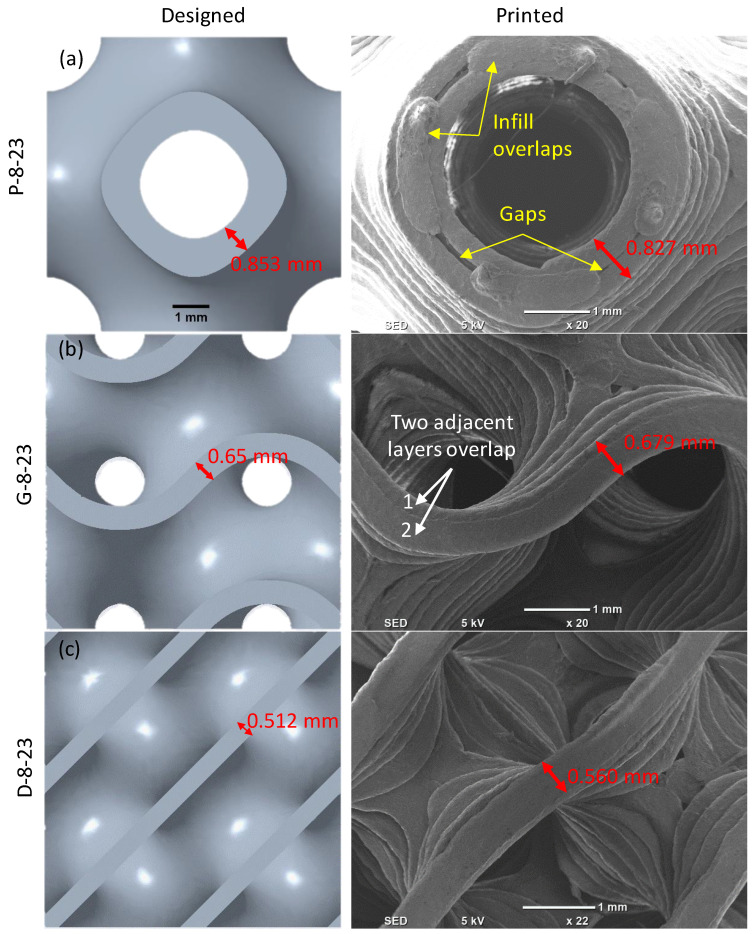
Designed and printed TPMS structures at 8 mm cell size and 23% RD: (**a**) Primitive, (**b**) Gyroid, and (**c**) Diamond.

The deviation of the as-built (actual) samples’ RDs to the corresponding CAD models is presented in Figure 5. With the exception of two extremes, D-6-30 and D-8-23, the relative densities of the as-built samples were generally in close agreement with the design values (maximum error ±8%). The main causes of these discrepancies are the presence of gaps between adjacent layers, infill overlapping, and the variation in wall thickness between the CAD and printed samples. Gaps occurred due to the inability to fill the small spaces between adjacent lines, resulting in reducing the RD. Figure 6a shows the presence of gaps in the Primitive structure at an 8 mm cell size and RD of 23% (P-8-23). Variation in wall thickness between actual and designed values may either increase or decrease the RD. This depends on whether the printed wall thickness is thicker or thinner than the corresponding CAD model. Figure 6 shows the variation of the wall thickness of Primitive, Gyroid, and Diamond structures at an 8 mm cell size and RD of 23%. In the case of P-8-23, the designed and actual wall thicknesses were 0.853 mm and 0.827 mm, respectively. The decrease in wall thickness could be the result of shrinkage. The measured RD of the P-8-23 was found to be 21.25%, which is lower than the designed RD (23%). In contrast, as illustrated in Figure 6b,c, the printed wall thickness of both the Gyroid and Diamond structures increased. This increase in wall thickness was caused by the overlapping of adjacent layers, as shown in Figure 6b,c. Overlapping occurs when the wall is thicker than the extrusion width. i.e., wall thickness is too thick to be printed in one line; hence, two lines are needed. As the overlap increases, the deviation increases, resulting in a considerable increase in RD, as in the case of D-8-23 (Figure 6c). A similar observation was noted for D-6-30 that showed a ~26% higher RD than the designed RD (30%).

The issues of gaps and overlapping are related to the FDM resolution and the designed wall thickness, which is a function of the cell size and the required RD. Thus, the RDs varied based on cell type, relative density, and cell size. To reduce these variations, unit cell size could be increased and/or a smaller size nozzle could be used, which is beyond the scope of this study. Printing TPMS designs with low RDs and a small cell size, which require thinner wall thicknesses, need to consider the FDM printing parameters, particularly extruding parameters (nozzle diameter and extrusion width).

### 3.2. Stress–Strain Response

**Figure 7:**
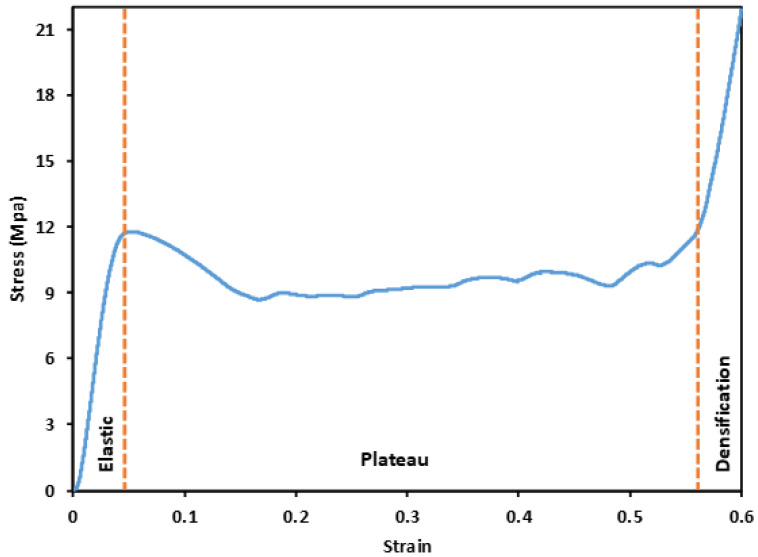
Typical compression behavior of a lattice structure; P-8-44 structure as an example.

For a typical lattice structure, stress–stain curves show three distinct regions: (1) an elastic compression region, where the stress increases linearly with strain during the elastic stage, demonstrating a linear stress–strain relationship; (2) a plateau region following the elastic region characterized by decreased stress and load-carrying capacity due to wall yield, plastic deformation, buckling, and fracture of the lattice walls; and (3) a densification region where collapsed walls and cells come into contact results in a significant increase in stress [36]. Figure 7 depicts a typical stress–strain curve for a Primitive lattice structure subjected to compression loading, exhibiting the three phases of linear elasticity, plateau stress, and densification.

**Figure 8:**
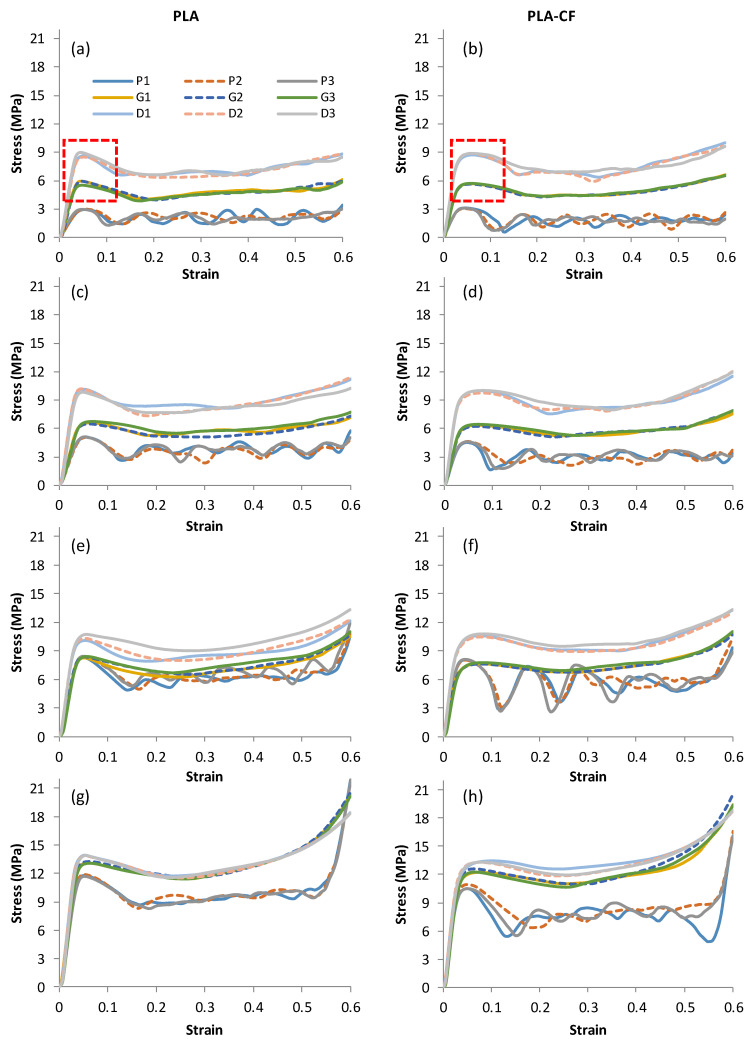
Stress–strain curves until 60% strain of Primitive, Gyroid, and Diamond structures at 8 mm cell size: (**a**,**b**) 23% RD, (**c**,**d**) 30% RD, (**e**,**f**) 37% RD, and (**g**,**h**) 44% RD. The red dashed boxes highlight the evident influence of CF incorporation on the plastic deformation.

**Figure 9:**
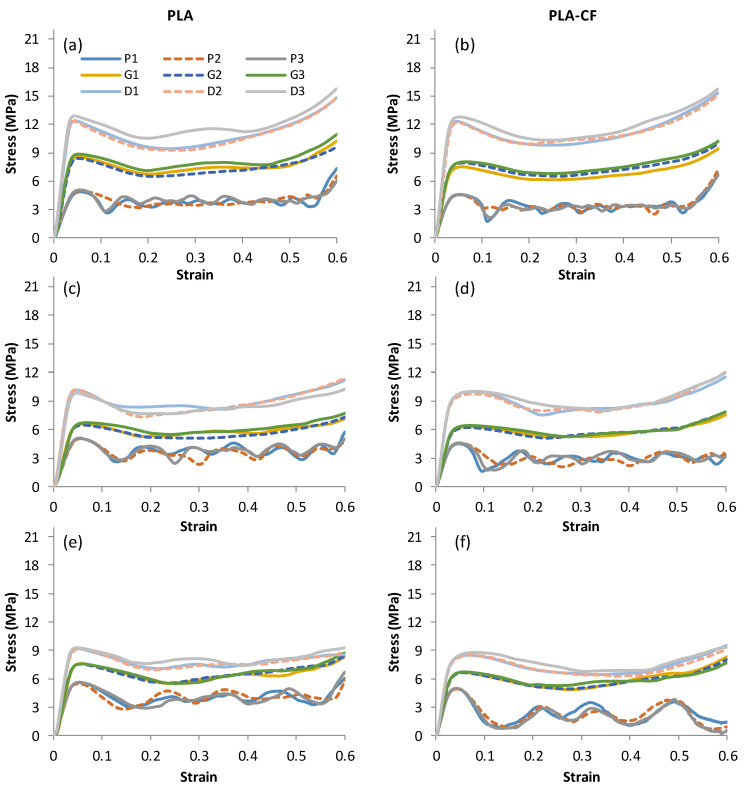
Stress–strain curves until 60% strain of Primitive, Gyroid, and Diamond structures at 30% RD: (**a**,**b**) 6 mm cell size, (**c**,**d**) 8 mm cell size, and (**e**,**f**) 12mm cell size.

Figure 8 and Figure 9 depict the stress–strain curves for the three replicates of the Primitive, Gyroid, and Diamond TPMS lattice structures at various relative densities (Figure 8) and unit cell sizes (Figure 9), as well as for the PLA and PLA + CF materials. The stress–strain plots show that the replicated samples are much closer to each other, particularly at the elastic part. Variations in cell topology, relative density, cell size, and material composition all had a considerable effect on the compressive responses. Except for the Primitive structure of PLA + CF material at 12 mm cell size (Figure 9f), all structures exhibit an initial linear elastic phase, followed by a plateau phase, and, finally, a densification phase for all considered conditions. It seems that D and G structures made of PLA + CF materials show more plastic deformation before the stress reduction compared to PLA samples, which show a sharp stress reduction after the elastic deformation, e.g., as circled with red rectangles in Figure 8a,b as an example.

Primitive structures exhibit repeating rise and fall in the stress–strain plot, which is an indication of the layer-by-layer pattern of deformation [29]. This can be noticed from the repeating rise and fall in the stress–strain curves, in which every wave represents a one-cell row. Gyroid and Diamond structures seem to be deformed uniformly, indicating consistent load bearing. Additionally, it is apparent that their deformation becomes more uniform with an increase in relative density and a decrease in cell size, minimizing waves in the stress–strain curves. This is a result of the high surface area and better material distribution within the geometry of the structure, which enhances the walls’ contact and minimizes the empty spaces between walls.

**Figure 10:**
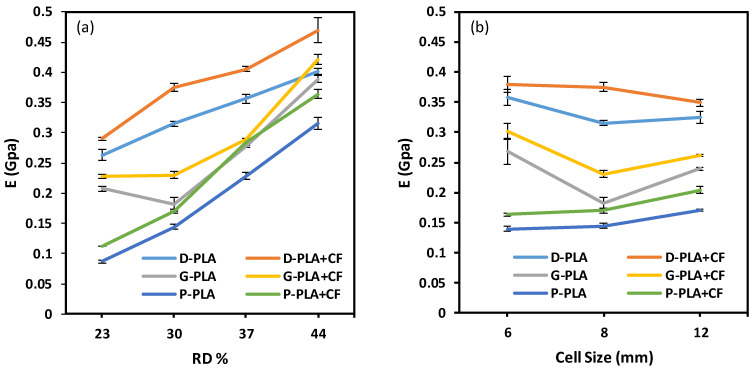
Compressive modulus of the Diamond, Gyroid, and Primitive structures at (**a**) different RDs while cell size remained at 8 mm and (**b**) different cell sizes while RD% remained at 30%.

**Figure 11:**
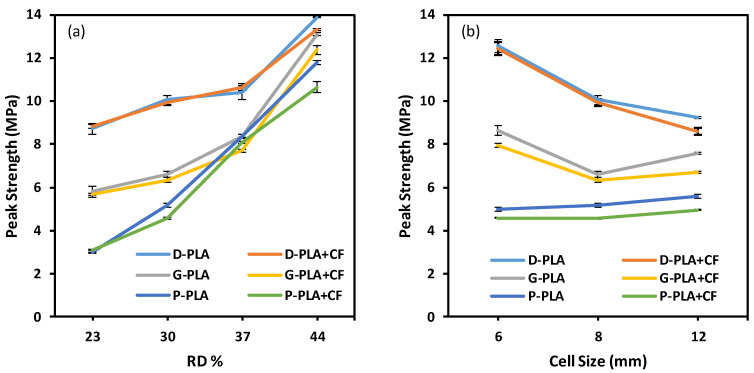
Peak strengths of the Diamond, Gyroid, and Primitive structures at (**a**) RDs while cell size remained at 8 mm and (**b**) different cell sizes while RD% remained at 30%.

The compressive characteristics of the three TPMS lattice structures are reported in Figure 10 and Figure 11. The variation, measured by standard deviation, in the three replicated experimental results, is shown by the error bars. The Diamond structure has the highest compressive strength and modulus for both materials (PLA and PLA + CF), followed by Gyroid and then Primitive. The results reveal that adding CF significantly increases the compressive modulus. Compared to PLA + CF structures, PLA structures show a higher peak strength than PLA + CF structures for the Gyroid and Primitive structures. The variation in the peak strength between PLA and PLA + CF structures, especially P and G structures, increases as the RD increases (see Figure 11a). This is due to the fact that P and G structures have much thicker wall thicknesses than Diamond structures. Furthermore, the P and G structures’ walls get thicker as RDs increase, resulting in more CF deposition, which leads to a reduction in their strength. Thus, at a low RD (23%), the compressive strength for both materials is close, as shown in Figure 11a. Primitive structures showed lower compressive modulus and strength compared to Gyroid and Diamond. It was also clear that relative density has an effect on both compressive strength and modulus, such that an increase in the relative density leads to an increase in the compressive modulus (Figure 10a) and strength (Figure 11a). It should be noted that the compressive modulus and peak strength of D-6-30 are greater than those of D-8-30 and D-12-30; this could be owing to the substantial variation between the actual and CAD RDs.

It is worth mentioning that the TPMS topology type (Diamond, Gyroid, and Primitive) substantially affects the compressive modulus and strength. This demonstrates that the mechanical properties of lattice structures are sensitive not only to the RD and material composition but also to the design type (P, G, and D) and unit cell size.

### 3.3. Compressive Deformation and Failure Mode Analysis

**Figure 12:**
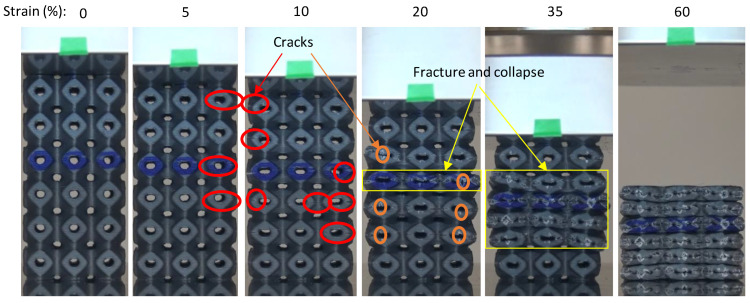
Typical deformation evolution of Primitive structure (P-8-44).

**Figure 13:**
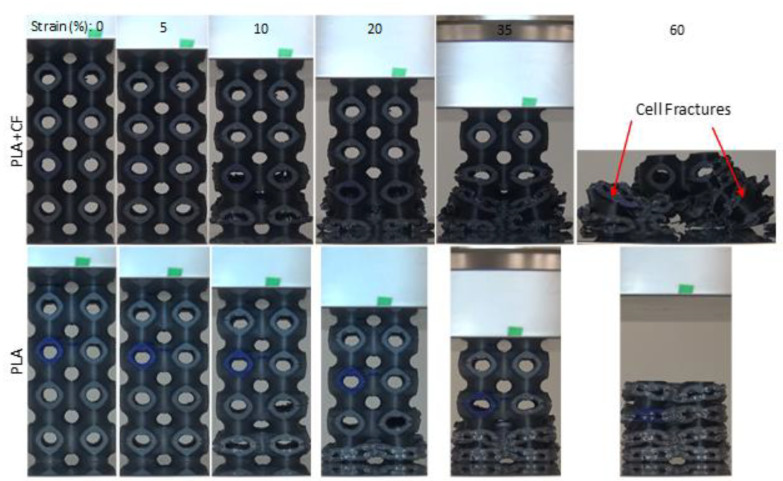
PLA + CF and PLA Primitive structures (P-12-30) deformation.

**Figure 14:**
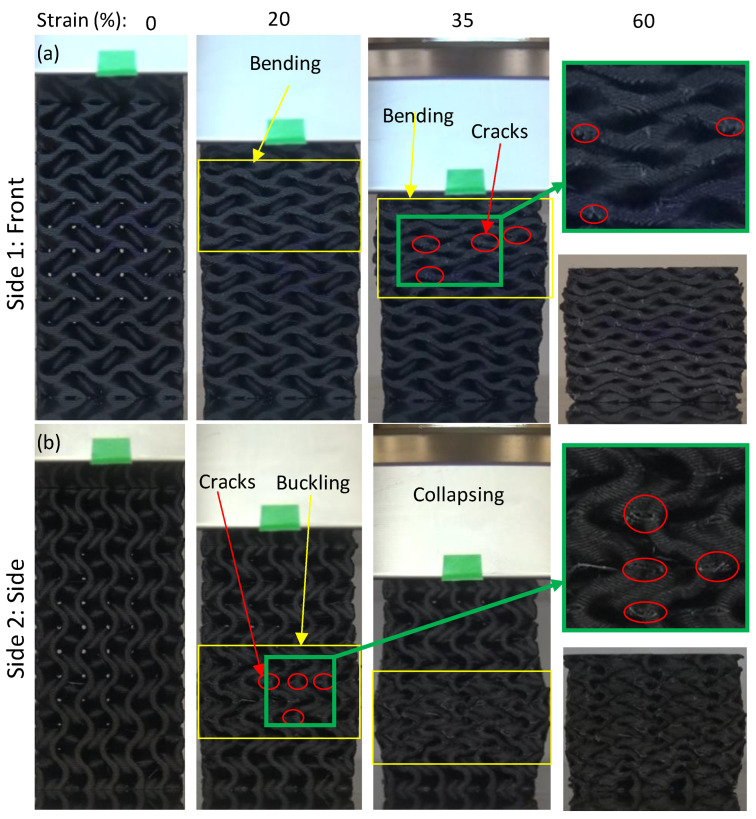
Typical deformation features of Gyroid structure (G-8-44 as an example) on two different sides: (**a**) front and (**b**) side.

**Figure 15:**
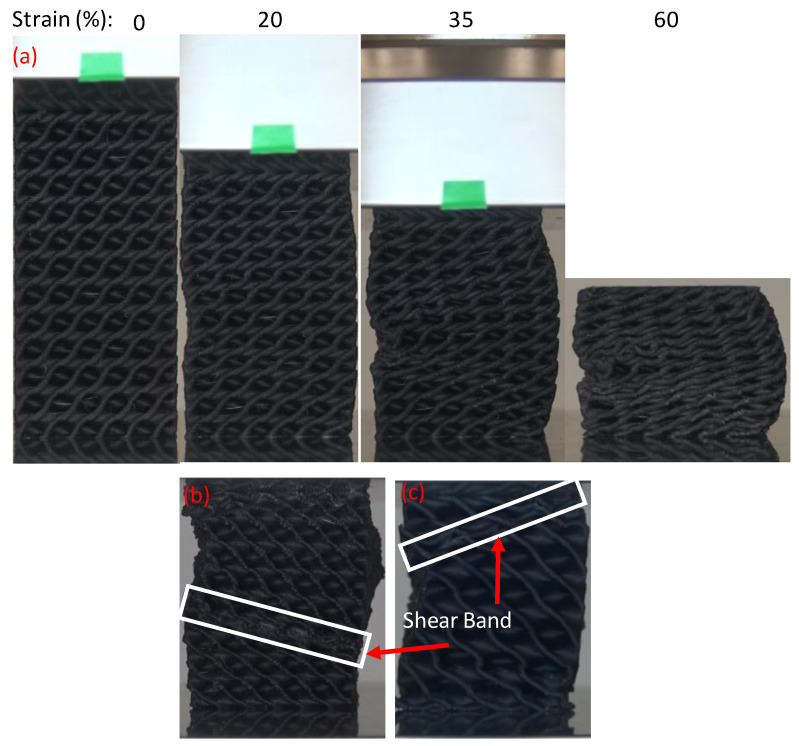
Deformation features of typical Diamond structures: (**a**) D-8-44, (**b**) shear band deformation of D-8-23 (at 35% strain), and (**c**) shear band deformation of D-12-30 (at 35% strain).

Deformation features of the samples are graphically illustrated in Figure 12, Figure 13, Figure 14 and Figure 15. Figure 12 shows that Primitive structures exhibit a layer-by-layer deformation pattern, which can also be noticed from the repeating rise and fall in the stress–strain curve in Figure 8 and Figure 9, in which every wave represents a one-cell row. Cracks between layers initiate the deformation, as indicated by the red circle. When the strain reaches 20%, damage and fracturing were clearly detected. As the compression continues, the walls’ fracture evolves gradually from layer to layer until all layers collapse at 60% strain, as shown in Figure 12. Fracturing and collapsing of the walls were clearly detected in all samples, with either PLA or PLA+CF. Cracks were also noticed across the building layers (vertical cracks), as circled with orange circles. At 23% RD, these cracks were not observed, which may be due to insufficient layer-to-layer bonding of the thinner walls that fracture before the across-layer cracks occur. In general, cracks always start between layers. The layer-by-layer deformation was uniform in the majority of the Primitive samples and the deformation starts occurring randomly either at the bottom, middle, or top in both PLA + CF and PLA samples. However, unlike other Primitive samples, instead of densification after layer-by-layer collapse, a complete failure of the P-12-30 lattice was observed for PLA + CF material, as shown Figure 13. Some unit cells were completely separated from the structures, while others remained intact. This is also evident from the stress–strain curve in Figure 9f, where the stress significantly dropped at the end of the test instead of densification.

Figure 14 depicts the Gyroid deformation during the compressive test from two different sides. Gyroid structure samples were deformed due to the bending of horizontal and angular walls (Figure 14a) and buckling of vertical walls (Figure 14b) with outward expansion. Figure 14 demonstrates that at 20% and 35% strain, cracks and fractures developed first as a result of buckling, which can be clearly detected in Figure 14 b. Furthermore, the other Gyroid structures showed similar failure behavior. Diamond structures exhibit a bending–torsion coupled deformation mode, as shown in Figure 15. Diamond structures showed the shear band formation due to bending–torsion coupled deformation, which is clearly detected at 35% strain of samples D-8-23 (Figure 15b) and D-12-30 (Figure 15c). Both Diamond and Gyroid structures have a tendency to accumulate deformation over time, which provides consistent load resistance.

### 3.4. Specific Energy Absorption

**Figure 16:**
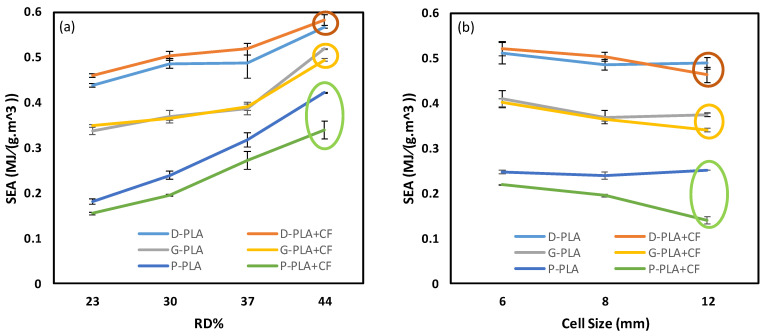
Specific energy absorption per volume of the Diamond, Gyroid, and Primitive structures at different: (**a**) unit cell sizes and (**b**) designed relative densities. Circles highlight the evident influence of the CF on the SEA at high RDs and larger cell size.

SEA is the most reliable method for evaluating a structure’s energy absorption capacity, since it accounts for the structure’s whole weight [34]. Figure 16 shows the SEA of the TPMS structures. It indicates that the addition of CF to PLA has a slight impact on the Diamond structures’ specific energy absorption, such that the incorporation of CF enhances the absorption energy capacity. However, a slight decrease in SEA was noticed for the 12 mm cell size, which similarly applies to the Gyroid and Primitive structures with the variation on the degree of influence. Regarding Gyroid structures, the addition of CF to PLA reduces the SEA at a high RD (44%) and larger unit cell size (12 mm). The primitive structure appears to be vulnerable to CF incorporation, resulting in a considerable decrease in specific energy absorption. Furthermore, when RD and cell size increased, the decrease in the SEA of PLA + CF-based Primitive structures was more pronounced. This could be attributed to the fact that thicker walls imply more CF extrusion, resulting in stiffer structures that can absorb less energy. Moreover, the increase in RD while keeping the cell size fixed or vice versa leads to an increase in wall thickness. Thus, in cases of high RD (44%) and cell size (12 mm), the influence of CF incorporation on the SEA of the Diamond, Gyroid, and Primitive structures was evident, as circled in Figure 16a,b. This suggests that there is a correlation between CF incorporation, cell topology, cell size, and RD. Furthermore, Primitive structures, with their thicker walls compared to the Gyroid and Diamond structures, are highly vulnerable to SEA reduction when incorporating CF (see Figure 16).

The specific absorbed energy of Diamond structures is more than that of Gyroid and Primitive structures, with Primitive structures having the least due to their layer-by-layer deformation mode [29]. RD has a proportionate effect on the specific energy absorption, such that an increase in RD results in an increase in specific energy absorption (see Figure 16a). The unit cell length has a significant effect on the specific energy absorption of Primitive structures made from PLA + CF material. As the cell length increases, the specific energy absorption decreases (Figure 16b).

To sum up, the mechanical properties and deformation patterns of the TPMS structures studied in this work were influenced by CF incorporation, design, and relative density parameters. The stress–strain curves of the TPMS structures under compression testing revealed different mechanical behavior depending on the considered parameters. For instance, the Primitive structures exhibited repeating rise and fall in the stress–strain plots (rise and fall numbers depend on the number of cell rows), while Gyroid and Diamond structures deformed uniformly. Furthermore, PLA + CF-based structures showed more plastic deformation before the stress reduction than PLA samples, which showed a sharp stress reduction after the elastic deformation. The mechanical response of the considered TPMS structures was enhanced by increasing relative density. Incorporating CF in the PLA + CF material significantly increases the compressive modulus in all TPMS structures. However, CF incorporation slightly decreased the compressive strength of both Gyroid and Primitive structures. Compared to the cell topology type, Primitive structures showed lower compressive modulus and strength compared to Gyroid and Diamond structures, which is similar to what was stated in [27]. Contrary to [27], Diamond structures showed the highest SEA compared to Gyroid and Primitive structures, which is consistent with the findings in [34]. Regarding deformation behavior, cell topology apparently influenced the deformation pattern. For instance, Primitive structures showed a layer-by-layer deformation pattern, Gyroid structures showed bending and buckling deformation, and Diamond structures showed bending–torsion coupled deformation [29].

Furthermore, the results in Figure 16 show the importance of comprehensively investigating materials, geometry, and relative density. As can be seen, the performance of the primitive structure, which does not exhibit improved SEA performance in the case of CF-reinforced composites, is good for other mechanical properties (e.g., compressive modulus). Similarly, the exact opposite phenomenon is observed for the Diamond structure, which exhibited a better SEA for PLA + CF and a worse SEA for pure PLA. This indicates that no single factor is optimal for mechanical characteristics, implying a correlation between material properties, design, and relative density parameters. Therefore, a careful selection of the appropriate combination of materials, design, and RD should be made based on the required mechanical properties.

## 4. Conclusions

In this study, three different TPMS lattice structures with varying relative densities and unit cell sizes were designed and fabricated using PLA and PLA + CF by the FDM process. The 3D-printed samples were subjected to a uniaxial compression test, and their mechanical performance, including compressive strength and modulus and specific energy absorption, were derived from the stress–strain curves. The following conclusions can be derived from the findings of this study:

- The measured RDs of the printed lattice structures were found to be in close agreement (max error ±8%) with the designed RD values. However, at a lower RD and smaller cell size, the Diamond topology showed a high deviation (~26%) from the designed RDs due to overlaps and FDM resolution leading to higher wall thickness.
- Like cell topology, cell size, relative density, and material composition considerably contribute to the different mechanical performances and quality of the 3D-printed lattice structures. For instance, a change in the compressive modulus (0.09 to 0.47 GPa), compressive strength (2.98 to 13.89 MPa), and specific energy absorption (SEA) (0.14 MJ/m^3^/g to 0.58 MJ/m^3^/g) was observed due to the influence of the investigated parameters.
- All structures showed higher compressive modulus for PLA + CF structures compared to the PLA material. However, the peak strength of Gyroid and Primitive designs for PLA + CF lattice structures was lower compared to that of PLA, and this effect became more pronounced at higher RDs.
- Comparing compressive modulus and strength, the Diamond structure outperforms the Gyroid and the Primitive structures. In most cases, the Primitive structures showed the worst performance.
- Compressive strength and energy absorption capacity were both slightly enhanced in most PLA + CF-based Diamond structures. On the contrary, PLA-based Gyroid and primitive structures showed better performance in terms of peak strength and specific energy absorption compared to PLA + CF-based structures.
- For all considered conditions, the Diamond lattice structure exhibits the highest specific energy absorption, followed by the Gyroid, and then the Primitive structure.

This work is limited to a single percentage (15%) of CF incorporation in PLA. The influence of different percentages of CF can be investigated on the lattice structures’ mechanical performance. Furthermore, the influence of FDM parameters, particularly extrusion parameters (nozzle diameter and extrusion width), can be taken into account to cope with the issues of overlapping and gaps to minimize the RDs’ deviations from the designed values.

## Figures and Tables

**Table 1:** Material compositions [31,32].

Material	CAS No.	Concentration, Weight %	
		PLA Resin	Carbon Fiber
PLA	9051-89-02	100	0
PLA + CF	(PLA Resin:9051-89-02, CF: 308063-67-4)	>80	<20

**Table 2:** Properties of PLA and PLA + F filaments [31,32].

Properties	PLA	PLA + CF
Density, g/cc	1.24	1.29
Tensile strength at break, MPa	56	48
Tensile Modulus, MPa	2865	4950
Tensile elongation at break, %	8	2

**Table 3:** Designed parameters of experimental tests.

No.	Lattice Designation	Cell Type	Cell Size *l* (mm)	RD %	No.	Lattice Designation	Cell Type	Cell Size *l* (mm)	RD %
1	P-8-23	P	8	23	10	P-8-44	P	8	44
2	G-8-23	G	8	23	11	G-8-44	G	8	44
3	D-8-23	D	8	23	12	D-8-44	D	8	44
4	P-8-30	P	8	30	13	P-6-30	P	6	30
5	G-8-30	G	8	30	14	G-6-30	G	6	30
6	D-8-30	D	8	30	15	D-6-30	D	6	30
7	P-8-37	P	8	37	16	P-12-30	P	12	30
8	G-8-37	G	8	37	17	G-12-30	G	12	30
9	D-8-37	D	8	37	18	D-12-30	D	12	30

**Table 4:** FDM Printing parameters.

Parameter	PLA and PLA + CF
Extruder temperature, °C	220
Bed temperature, °C	65
Nozzle diameter, mm	0.4
Printing speed, mm/s	45
Layer height, mm	0.2
Infill, %	100

**Table 5:** Relative densities of the Diamond, Gyroid, and Primitive at different unit cell sizes and designed relative densities.

No.	Design	As-Built RD (RD% ± SD)		No.	Design	As-Built RD (RD% ± SD)	
		PLA	PLA + CF			PLA	PLA + CF
1	P-8-23	21.26 ± 0.47	21.25 ± 0.25	10	P-8-44	43.48 ± 0.07	42.51 ± 0.33
2	G-8-23	24.90 ± 0.22	24.59 ± 0.18	11	G-8-44	44.99 ± 0.15	44.15 ± 0.66
3	D-8-23	29.09 ± 1.01	27.88 ± 0.11	12	D-8-44	42.65 ± 0.31	42.35 ± 0.17
4	P-8-30	28.50 ± 0.27	28.11 ± 0.13	13	P-6-30	28.82 ± 0.06	28.08 ± 0.09
5	G-8-30	28.91 ± 0.34	28.51 ± 0.28	14	G-6-30	32.67 ± 0.13	31.76 ± 0.09
6	D-8-30	32.36 ± 0.26	31.18 ± 0.06	15	D-6-30	37.91 ± 0.49	36.84 ± 0.3
7	P-8-37	37.07 ± 0.14	37.14 ± 0.23	16	P-12-30	28.59 ± 0.2	28.55 ± 0.07
8	G-8-37	35.20 ± 0.48	34.91 ± 0.24	17	G-12-30	30.48 ± 0.07	30.18 ± 0.07
9	D-8-37	35.62 ± 0.59	35.36 ± 0.53	18	D-12-30	28.50 ± 0.09	28.11 ± 0.09

## Data Availability

The data presented in this study are available on request from the corresponding author.
